# Measuring the Coverage of the HL7® FHIR® Standard in Supporting Data Acquisition for 3 Public Health Registries

**DOI:** 10.1007/s10916-023-02033-z

**Published:** 2024-02-08

**Authors:** Manju Bikkanuri, Taiquitha T. Robins, Lori Wong, Emel Seker, Melody L. Greer, Tremaine B. Williams, Maryam Y. Garza

**Affiliations:** 1 Department of Population Health Sciences, University of Texas Health Science Center at San Antonio, San Antonio, TX USA; 2 Department of Biomedical Informatics, University of Arkansas for Medical Sciences, 4301 W Markham St., #782, Little Rock, AR 72205 USA; 3 Health Data Innovation Program, Keck Medicine of USC, Los Angeles, CA USA

**Keywords:** HL7 FHIR, Registries, Public Health, Data Elements, Interoperability, Real-world data

## Abstract

With the increasing need for timely submission of data to state and national public health registries, current manual approaches to data acquisition and submission are insufficient. In clinical practice, federal regulations are now mandating the use of data messaging standards, i.e., the Health Level Seven (HL7^®^) Fast Healthcare Interoperability Resources (FHIR^®^) standard, to facilitate the electronic exchange of clinical (patient) data. In both research and public health practice, we can also leverage FHIR^®^ ‒ and the infrastructure already in place for supporting exchange of clinical practice data ‒ to enable seamless exchange between the electronic medical record and public health registries. That said, in order to understand the current utility of FHIR^®^ for supporting the public health use case, we must first measure the extent to which the standard resources map to the required registry data elements. Thus, using a systematic mapping approach, we evaluated the level of completeness of the FHIR^®^ standard to support data collection for three public health registries (Trauma, Stroke, and National Surgical Quality Improvement Program). On average, approximately 80% of data elements were available in FHIR^®^ (71%, 77%, and 92%, respectively; inter-annotator agreement rates: 82%, 78%, and 72%, respectively). This tells us that there is the potential for significant automation to support EHR-to-Registry data exchange, which will reduce the amount of manual, error-prone processes and ensure higher data quality. Further, identification of the remaining 20% of data elements that are “not mapped” will enable us to improve the standard and develop profiles that will better fit the registry data model.

## Background

As more federal regulations require reporting to clinical data registries, the role of registries in clinical practice, research, and public health has never been more significant. Registries, often referred to as clinical data registries, patient registries, public health registries, or disease registries, provide a rich source of healthcare information that can be leveraged across the healthcare enterprise to improve patient and population health outcomes. For decades, registries have offered researchers “the opportunity to incorporate patient, provider, and health system perspectives while collecting and analyzing health information and outcomes” [1].

Registries rely heavily on the submission of real-world data^1^ (RWD) ‒ observational data obtained from real-world settings (e.g., patient reported outcomes, mobile health devices) and routine clinical practice (e.g., clinical encounters, electronic medical records) pertaining to patient health status and health-related activities taken by or delivered to a patient, as opposed to data derived from controlled (i.e., clinical research) settings [2–5]. RWD are valuable sources of information that hold great potential in generating robust, real-world evidence^2^ to provide clinically-rich insights, support regulatory decision-making, and, ultimately, improve patient outcomes. The US Food and Drug Administration (FDA) [5], National Institutes of Health (NIH) [6, 7], and Centers for Disease Control and Prevention (CDC) [8] have all issued calls to action to healthcare and public health professionals to identify ways to leverage RWD in daily practice and research. As registries are large repositories of RWD, they are potential goldmines of valuable patient information.

Registries often rely on manual medical record abstraction (MRA) to extract electronic health record (EHR) data on a particular disease, condition, or exposure. They can act as a primary database of aggregate, longitudinal health information for use in clinical research studies and to support public health initiatives. However, manual approaches have had a negative impact on cost and quality, primarily due to the complexities associated with data collection, or abstraction, and mapping of the data to fit the registry data model [9]. Improvements to systems interoperability ‒ through the use of standards-based methods ‒ have the potential to address these issues by automating part or all of the data collection process.

The Health Level Seven (HL7®) Fast Healthcare Interoperability Resources (FHIR®) standard [10] is an international data exchange (messaging) standard adopted by EHR vendors [11–13] in response to the 21st Century Cures Act [14, 15]. Use of FHIR® allows for near-real-time data collection directly from EHRs to electronic data capture (EDC) systems, such as registry databases. However, to our knowledge, robust and generalizable implementations and evaluations of the Registry-to-FHIR use case have not been conducted [16–18]. Assessment of data quality and data collection burden were identified as systematic weaknesses by previous studies [16–18]. As a potential source of RWD for clinical studies and national benchmarking programs, a rigorous assessment and comparison of performance on data quality, specifically the level of data element concordance between FHIR® and clinical data registries, is urgently needed to advance the use of RWD.

We anticipate the use of FHIR® will streamline the registry data acquisition and submission processes, which will, in turn, improve the overall data quality and reduce time and effort requirements, and costs associated with current collection methods. Although, in order to accurately measure the potential improvements, we must first evaluate the coverage of the standard and its ability to support the needs of the registry use case. Therefore, we evaluated the HL7® FHIR® standard data elements against those collected by three nationally recognized registries to determine the total number of data elements supported by FHIR® and eligible for direct, electronic data exchange.

## Methods

The primary objective of this study was to measure the extent to which the FHIR® standard accurately represented the data elements required to support data acquisition for and submission to three nationally recognized registries. The study team selected three registries to which the University of Arkansas for Medical Sciences regularly supplies data ‒ the Trauma, Stroke, and National Surgical Quality Improvement Program (NSQIP) registries (Table 1). We consulted the full list of registries located on the Arkansas Department of Health website and identified the most popular registries with the broadest, national presence for generalizability. Data dictionaries^3^ for each registry were publicly available and used for mapping to the FHIR® standard.

**Table 1:** Registries of Interest

Registry	Population	Clinical Focus	State Registry	National Registry
Trauma	Adult / Pediatric	Trauma, Injury	Arkansas Trauma Registry	National Trauma Data Bank (NTDB®)
Stroke	Adult	Acute Stroke Care	Arkansas Stroke Registry	National Acute Stroke Program
NSQIP	Adult / Pediatric	Surgery, Surgical Complications	‒	National Surgical Quality Improvement Program (NSQIP)

Registry: name of the registry of interest; Population: population supported by the registry (adult vs. pediatric vs. both); Clinical Focus: clinical focus or domain area of interest of the registry; State Registry: name of the state-based registry program associated with the registry of interest; National Registry: name of the nationally recognized registry program

A systematic mapping approach, developed by Garza and colleagues [19], was used to conduct the Registry-to-FHIR data element mappings. Registry data elements were mapped using the latest version (at the time this work was started) of the base standard ‒ the HL7® FHIR® Version Release 4b (FHIR® R4b) standard [10] ‒ the FHIR® US Core Profiles, Release 4 (US Core R4), and the US Core Implementation Guide STU4 [20].

Two independent team members conducted the mappings between the registry case report forms (CRFs) and the FHIR® standard, hereinafter referred to as the Registry-to-FHIR mappings. Mappers first identified the appropriate FHIR® resource for a registry data element, and then identified the appropriate data element(s) within the resource that best fit the registry data element based on its definition. Permissible values and data type were considered in situations where those were not extensible in the FHIR® resource. In other words, data elements that were flexible enough, were mapped to FHIR® resources even if the example resource coding did not match the registry values perfectly. Briefly, for coded data elements, those with permissible value lists (e.g., controlled terminologies, code sets, or value sets) FHIR® offers different binding strengths that are “associated with various degrees of flexibility as to how closely the value set should be followed”: Required, Extensible, Preferred, Example [21].

In situations where our data element captured more values than those in a resource using a Required binding, we would need to determine if we would be able to fit our code list by mapping values to the ‘other’ option ‒ accepting the information loss ‒ or else consider this as “Not Available in FHIR” for our use case. However, in situations where the resource used Extensible bindings, if the concepts in the registry permissible value set do not match at all, alternate codes can be used instead, and we could consider this as “Available in FHIR” so long as the data element definition matches the definition in our use case. Even more flexibility is available for data elements with Preferred and Example bindings, which are completely optional. For example, Procedure.code uses ‘SNOMED CT’ as an example binding, which gives us the option to use SNOMED CT, but does not limit us to that code set. Instead, we could use CPT codes and consider this data element as “Available in FHIR” so long as the definition matches that of our use case.

Adjudications of the Registry-to-FHIR mappings were performed by three members of the research team (2 mappers, 1 principal investigator) to achieve consensus prior to the analysis to address any conflicts in the mappings. The principal investigator performed a cursory review of the two mappings, consolidated the mappings into a singular dataset, and identified those that were not in agreement. The adjudication team then discussed the conflicting data elements and determined the final mapping for each through a consensus-driven approach. In the event consensus could not be reached, the principal investigator made the final decision after seeking input from the HL7® community.

As a result of the Registry-to-FHIR mappings, data elements for each registry were categorized into two groups: “Available in FHIR” and “Not Available in FHIR”. These categories were used to calculate the actual coverage of the standard and a percentage for each category. Following the same criteria for coverage as prior mapping work by Garza and colleagues [22], we considered a data element as *Available in FHIR* “…if (1) it has a corresponding data element available within one of the FHIR® resources or (2) if the data can be captured or derived using a combination of data elements across one or more resources.” This represented the transformation between FHIR® resources and the corresponding registry. The FHIR® coverage measure was calculated as a percentage (total data elements *Available in FHIR* vs. total data elements overall).

In addition, in an effort to identify the clinical domain areas with the least and most coverage, we analyzed coverage across the different domain areas for each registry. We further analyzed the complexities of the mappings by reviewing the total number of resources needed to fully capture the context specific to a particular registry data element. For example, ‘Date of Birth’ would be considered a one-to-one mapping (least complex), as the singular registry data element would map directly to the ‘birthDate’ data element within the Patient Resource. However, ‘CT Date/Time’ would be slightly more complex, as it would require mapping to the Procedure Resource to capture the date/time component, but also require mapping to (or reference to) the Encounter Resource to ensure that the Procedure occurred during the specified encounter. In this case, at least three FHIR® data elements would be required: Procedure.code (to specify the type of procedure as a ‘CT’), Procedure.occurrence[x] (to capture the date/time component), and Procedure.encounter (to reference back to the Encounter Resource and ensure this procedure occurred during our encounter of interest).

## Results

Originally, we started with 792 total data elements across all three registries (Trauma: 288, Stroke: 230, NSQIP: 274). Details pertaining to the data elements collected for each registry (name, definition, data type, etc.) can be found in the Supplemental Files. For the Stroke registry, we decided to exclude 24 data elements that were listed as ‘Optional’ because the data element name was vague and did not allow for mapping (i.e., Field 1, Field 2…, Field n). These were listed as ‘N/A’ and removed from the total, leaving us with 206 Stroke data elements, and a new total set of 768 total data elements. On average, 80% (*n* = 613) were *Available in FHIR* and 20% (*n* = 155) were *Not Available in FHIR*.

**Fig. 1:**
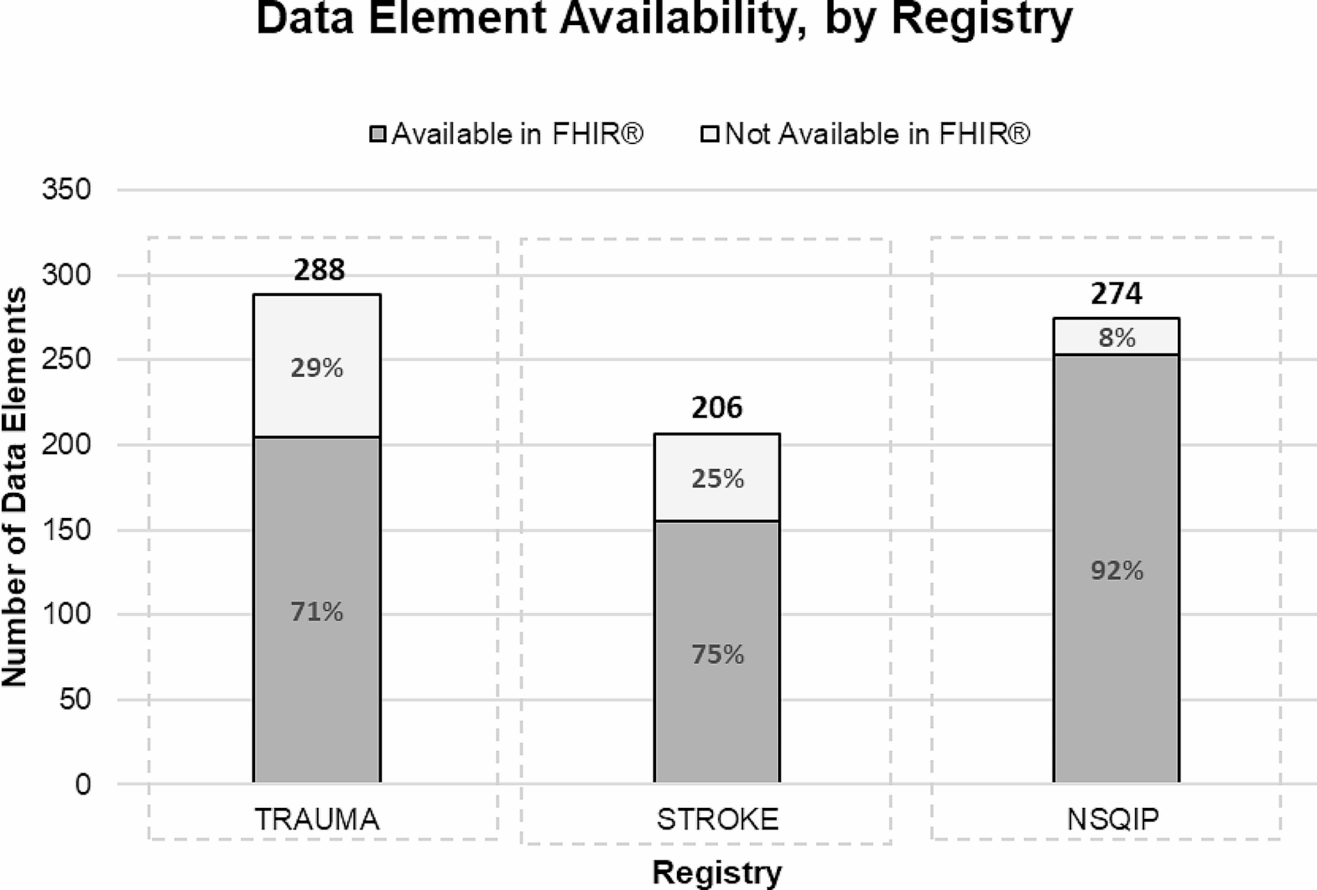
FHIR Mapping Rates for Data Elements By Registry. FHIR: Fast Healthcare Interoperability Resources; NSQIP: National Surgical Quality Improvement Program

When we reviewed each registry individually, we obtained the following results (Fig. 1). Of the 288 data elements in the Trauma registry, 71% (*n* = 205) were *Available in FHIR* and 29% (*n* = 83) were *Not Available in FHIR*. Of the 206 Stroke registry data elements, 75% (*n* = 155) were *Available in FHIR* and 25% (*n* = 51) were *Not Available in FHIR*; and of the 274 NSQIP registry data elements, 92% (*n* = 253) were *Available in FHIR* and 8% (*n* = 21) were *Not Available in FHIR*. The inter-annotator agreement for each of the mappings resulted in a Cohen’s kappa of 82%, 78%, and 72%, respectively.

When we evaluated the overlap between the three registries (data elements were similar enough to be considered as “in common”), the Trauma and Stroke registries had 45% (129 out of 288) in common, compared to the Trauma and NSQIP registries, which had 58% (167 out of 288) in common. The Stroke and NSQIP registries had 72% (138 out of 274) of data elements in common. Table 2 depicts the registry data elements by domain area, comparing each of the three registries to one another, and then displays how this compares to the FHIR® resources. For example, both the Trauma and Stroke registries collected a relatively small percentage of administrative data (Trauma: 22% versus Stroke: 8%), as compared to the NSQIP registry which only captured clinical data elements (i.e., Demographics, Diagnoses, Encounters, etc.). Trauma and Stroke were similar in their domain breakdown, having a majority of data elements fall within the “Encounter” or “Hospitalization” domain (34% and 70%, respectively), which translated primarily to the Encounter Resource in FHIR®.

**Table 2:** Registry Data Element Breakdown by Clinical Domains by Registry

CLINICAL DOMAIN AREAS						
DOMAINS	TRAUMA (*N* = 288)		STROKE (*N* = 230)		NSQIP (*N* = 274)	
	count	percent	count	percent	count	percent
Administrative	63	22%	18	8%	-	-
Demographics	22	8%	9	4%	6	2%
Diagnosis	33	11%	4	2%	116	42%
Encounter	99	34%	162	70%	20	7%
Labs	3	1%	-	-	26	10%
Observation	31	11%	3	1%	3	1%
Procedure	3	1%	-	-	99	36%
Provider	5	2%	-	-	-	-
Transfer Data	29	10%	-	-	-	-
Social History	-	-	-	-	1	1%
Vital Signs	-	-	-	-	3	1%
Miscellaneous	-	-	34	15%	-	-

NSQIP: National Surgical Quality Improvement Program. N: refers to the total number of data elements captured within the corresponding registry. Count: refers to the number of data elements captured within the corresponding domain. Percent: refers to the number of data elements captured within the corresponding domain divided by the total number of data elements captured within the corresponding registry (N)

After evaluating for the most common FHIR® resources to which the registry data elements mapped (Table 3), we found that, for the Trauma registry, most of the data elements were captured within three resources: Observation (31%), Condition (23%), and Encounter (16%), having mapped across 10 different resources in total. The data elements from the Stroke registry were spread across a wider variety of resources (16 total resources), including Observation (16%), Procedure (15%), Encounter (10%), Condition (10%), Medication Request (9%), Service Request (9%), and Medication Administration (6%). The data elements from the NSQIP registry mapped to the fewest number of resources at 6 total, which included Condition (32%), Procedure (29%), Encounter (24%), Observation (12%), Patient (2%) and Medication Statement (1%).

**Table 3:** FHIR® Resources by Registry: Coverage of Data Elements *Available in FHIR*

FHIR® RESOURCES						
RESOURCES	TRAUMA (*n* = 205)		STROKE (*n* = 155)		NSQIP (*n* = 253)	
	count	percent	count	percent	count	percent
Condition	47	23%	15	10%	81	32%
Coverage	-	-	1	1%	-	-
Device	3	1%	-	-	-	-
DiagnosticReport	-	-	5	3%	-	-
Dosage	-	-	1	1%	-	-
Encounter	32	16%	16	10%	61	24%
EpisodeOfCare	5	2%	5	3%	-	-
MedicationAdministration	12	6%	10	6%	-	-
MedicationKnowledge	-	-	9	6%	-	-
MedicationRequest	-	-	14	9%	-	-
MedicationStatement	2	1%	6	4%	2	1%
NutritionOrder	-	-	1	1%	-	-
Observation	64	31%	25	16%	31	12%
Patient	17	8%	9	6%	5	2%
Practitioner	1	0%	-	-	-	-
Procedure	22	11%	23	15%	73	29%
ResearchSubject	-	-	1	1%	-	-
ServiceRequest	-	-	14	9%	-	-

FHIR: Fast Healthcare Interoperability Resources; NSQIP: National Surgical Quality Improvement Program. n: refers to the total number of data elements Available in FHIR within the corresponding registry. Count: refers to the number of data elements Available in FHIR captured within the corresponding Resource. Percent: refers to the number of data elements Available in FHIR captured within the corresponding Resource divided by the total number of data elements Available in FHIR within the corresponding registry (n)

## Discussion

The coverage of the HL7® FHIR® standard was measured against three nationally recognized registries to discover if data acquisition would be well supported by the internationally recognized FHIR® standard. Broadly, we found substantial coverage of the registry data elements in FHIR®, which supported between 71 and 92% of data elements across the three registries reviewed. These findings indicate that, if automated or semi-automated data extraction processes are applied to support data collection for these registries, on average, nearly 80% of the data elements could be extracted directly from the source system (i.e., EHR).

Furthermore, we see that, between the three registries evaluated, on average, nearly half of the data elements were shared across the registries. For an institution that participates in all three registries, it means that the EHR-to-FHIR mapping would only be required once for those shared data elements, and the mappings could be reused for other registries, ultimately reducing the mapping burden. This can be extrapolated further to other registries and public health initiatives in general that would require RWD from an institution’s EHR.

Another major benefit implied by these results is the reduction in manual abstraction and submission of these data elements to the registry. This would substantially limit human-related errors inherent in manual processes, such as MRA. Prior research by Garza and colleagues [23] has demonstrated the high and highly variable error rates associated with MRA (70 ‒ 2,784 errors per 10,000 fields). By reducing the number of data elements requiring manual processing ‒ for example, from 288 to 83 for the Trauma registry, from 230 to 51 for Stroke, or from 274 to 21 for NSQIP ‒ the amount of potential error also decreases. Further, the ability to utilize automated (or semi-automated) approaches also has the potential to decrease the amount of time that is currently devoted to managing MRA for these registries, which will allow for more efficient resource allocation. For future consideration, as FHIR-based solutions are implemented and total automation is sought, thresholds must be established and managed to identify acceptable limits of errors, proportionate to increasing higher levels of automation, as reliance on MRA is reduced.

Overall, the results were consistent with prior, limited studies of FHIR® integration [22, 24]. These results are also consistent with FHIR’s 80/20 rule, in that the resources have been designed to support a more general or common set of data requirements across many use cases. It is known that there will be gaps in the standard (the base standard aims to satisfy 80% of the interoperability needs), but these gaps are not always clear. Our work has helped to clarify the domains or types of data elements that are not currently supported by the base standard. With that knowledge we can determine the best course of action for addressing gaps in mapping. Several options exist: (1) do nothing and move forward with 80% coverage or 80% automation and continue to utilize manual approaches for the remaining 20% not covered by the standard; (2) extend the standard through the development of extensions or profiles that could potentially address the 20% gap, where appropriate; or (3) consider how the source system currently captures and stores the data, and determine if there are more efficient ways to do so that would allow for mapping to a standard. In posing these options, we are not recommending changing the base standard, as this becomes too nuanced and burdensome when expanded to the wide variety of registries in existence. Instead, further exploration is necessary to determine whether or not certain profiles or extensions would be appropriate and beneficial. We also recommend that sites and registry networks take inventory of the current set processes in place to determine if opportunities exist for improvement that would result in downstream benefits.

### Limitations

We would like to acknowledge that, the FHIR® mappings conducted, while performed using a systematic approach, were done so to the base standard and US Core extensions as written, and we did not test the mappings against the UAMS EHR. Therefore, we cannot say with 100% certainty that the total number of data elements *Available in FHIR* is exactly what is currently available at our (or other) institution(s). However, the rates identified as part of this study are optimistic rates, as they imply that the base standard is fully implemented at a site as designed. In most cases, the version of the standard used at sites is dependent upon the maturity level^4^ of the FHIR® resource and the particular type and version of the EHR implemented at the site.

Furthermore, the amount and type of RWD captured in source systems (e.g., EHRs) may vary across patients, physicians, and source systems. Accordingly, even if data elements from an EHR are available in FHIR, that does not necessarily correspond to data being available in the EHR to exchange via FHIR. In other words, there will be natural variability in the rates of data availability across sources.

## Conclusion

In our evaluation of Registry-to-FHIR mapping for three nationally recognized public health registries, we found that a majority of the data elements were available in FHIR® (71 ‒ 92%). We posit that registries requiring similar data elements to the registries evaluated here are likely to fare much the same as the three reviewed here, with nearly 80% coverage, on average. As informatics advances as an independent scientific discipline, FHIR-enabled solutions for research and public health will expand and move beyond examining the feasibility of data pipelines between clinical information systems and registries and reduce reliance on error-prone, manual abstraction processes.

## Data Availability

All data generated or analyzed during this study are included in this published article and its supplementary information files.

## Abbreviations

FDA: Food and Drug Administration
NIH: National Institutes of Health
CDC: Centers for Disease Control and Prevention
RWD: Real-World Data
RWE: Real-World Evidence
MRA: Medical Record Abstraction
EHR: Electronic Health Record
NQRN: National Quality Registry Network
HL7®: Health Level Seven
FHIR®: Fast Healthcare Interoperability Resources
NSQIP: National Surgical Quality Improvement Program
NTDB®: National Trauma Data Bank
CRF: Case Report Form

## Footnotes

1 NIH Definition for Real-World Data (RWD): *refers to data relating to patient health status and/or the delivery of health care routinely collected provided outside of a research setting. RWD may be collected from a variety of sources including the patient, clinician, registries, or electronic medical records.*

2 NIH Definition for Real-World Evidence (RWE): *refers to the clinical evidence regarding the usage and potential benefits or risks of a medical product derived from analysis of real-world data.*

3 Data Dictionaries: (1) Trauma ‒ (a) https://www.healthy.arkansas.gov/images/uploads/pdf/ATR_2023_Data_Dictionary_-_part_1.pdf, (b) https://www.healthy.arkansas.gov/programs-services/topics/arkansas-trauma-registry-atr; (2) Stroke ‒ (a) https://www.heart.org/-/media/Files/Professional/Quality-Improvement/Get-With-the-Guidelines/Get-With-The-Guidelines-Stroke/Stroke--Diabetes-CRFJuly21.pdf, (b) https://www.heart.org/-/media/Files/Professional/Quality-Improvement/Get-With-the-Guidelines/Get-With-The-Guidelines-Stroke/Stroke-30Day-FollowUp-Form-CRF.pdf; (3) NSQIP ‒ https://www.facs.org/media/yaol5yoj/nsqip_puf_userguide_2020.pdf.

4 Maturity Level: In standards development, the maturity level of a data element or resource is based on the level of testing or review undertaken as part of the formalized standard balloting process and corresponds with overall stability of the element or resource.
